# Publisher Correction: Upper critical field and superconductor-metal transition in ultrathin niobium films

**DOI:** 10.1038/s41598-021-87426-1

**Published:** 2021-04-13

**Authors:** Iryna Zaytseva, Aleksander Abaloszew, Bruno C. Camargo, Yevgen Syryanyy, Marta Z. Cieplak

**Affiliations:** Institute of Physics, Polish Academy of Sciences, Aleja Lotników 32/46, 02668 Warsaw, Poland

Correction to: *Scientific Reports* 10.1038/s41598-020-75968-9, published online 04 November 2020

This Article contains an error in Figure 1 where all data points in the right inset of panel (g) are missing.

The correct Figure 1 appears below.

**Figure 1:**
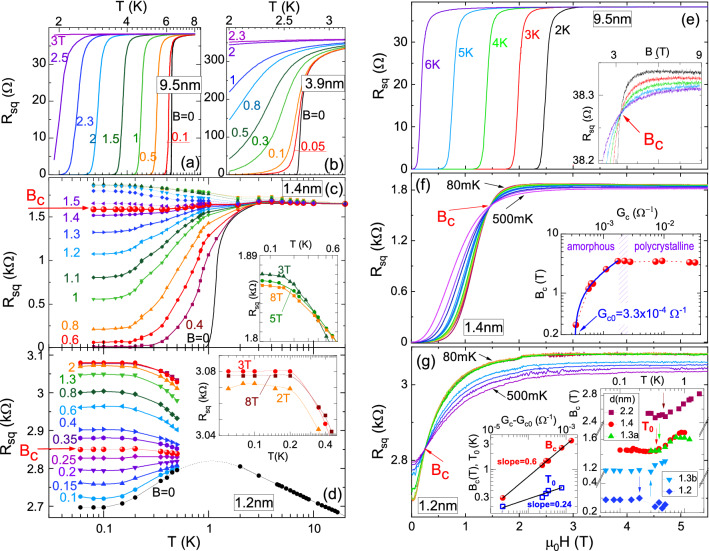
*R*_*sq*_ versus *T* for various *B* as labeled near the curves (in teslas) (**a**–**d**), and *R*_*sq*_ versus *B* at various *T* as labeled near the curves (**e**–**g**), for films with various *d*: (**a**) 9.5 nm, (**b**) 3.9 nm, (**c**) 1.4 nm, (**d**) 1.2 nm, (**e**) 9.5 nm, (**f**) 1.4 nm, (**g**) 1.2 nm. Red arrows indicate *B*_*c*_. Insets in (**c**) and (**d**): regions of negative MR on the expanded scale. Inset in (**e**): expanded region near *B*_*c*_. Inset in (**f**): *B*_*c*_ versus *G*_*c*_ (measured at the lowest *T*). Blue curve shows the fit described in the text, *B*_*c*_ = *A*(*G*_*c*_ − *G*_*c*0_)^p^. Right inset in (**g**): *B*_*c*_ versus *T* for several amorphous films. The arrows indicate *T*_0_, below which *B*_*c*_ is constant. Left inset in (**g**): *B*_*c*_ and *T*_0_ versus *G*_*c*_ − *G*_*c*0_ on a double logarithmic scale. The lines have slopes of 0.6 and 0.24, respectively.

