# Supplementary material for: Upper critical field and superconductor-metal transition in ultrathin niobium films
Source: Sci Rep. 2020 Nov 4;10:19062. doi: 10.1038/s41598-020-75968-9 (PMC7642399; doi:10.1038/s41598-020-75968-9)
Supplement: Supplementary file 1 — Supplementary Information. [file 41598_2020_75968_MOESM1_ESM.pdf]

Upper critical field and superconductor-metal transition in ultrathin niobium films

Iryna Zaytseva[1], Aleksander Abaloszew[1], Bruno C. Camargo[1], Yevgen Syryanyy[1], and Marta Z. Cieplak[1]

[1]Institute of Physics, Polish Academy of Sciences, Aleja Lotnikow 32/46, PL-02668 Warsaw, Poland

SUPPLEMENTARY INFORMATION

Film preparation and characterization

The films were made by magnetron sputtering at room temperature on glass substrates, with niobium sandwiched between Si-buffer layers to prevent Nb oxidation[1]. High-resolution transmission electron microscopy and X-ray diffraction studies[1] indicate that the films with $d < 3.3$ nm are amorphous, without any indications of gross structural inhomogeneity. The thickness of the films is quite uniform, without any traces of polycrystalline grains. On the other hand, all polycrystalline films contain a uniformly thin layer (of thickness of about 1.5 nm) of amorphous Nb at the Nb/Si interface, formed in the initial stage of the Nb deposition. Note that this amorphous layer is thinner than 3.3nm. This suggests that, when d exceeds 3.3 nm during Nb deposition, the polycrystalline grains begin to form, and part of the amorphous layer at the interface recrystallizes. X-ray Photoelectron Spectroscopy of the thinnest films is consistent with small admixture of Si ions (5 to 10 at.%) into Nb layer closest to the interface[2]. The change of sign of the Hall coefficient is observed, from positive in thick polycrystalline films, to negative in thinnest films at the lowest temperatures; it is most likely related to strong surface scattering of hole carriers[1].

For magnetoresistance measurements (in magnetic field perpendicular to the film plane) the films were cut into 5×5 mm^2 size and they were lithographically patterned into "Hall bar" structure, with the current path 2 mm long and 200 μm wide, as shown in Fig. S1(a) (current contacts are marked A-B). In order to prevent sample heating due to noise or excessive current the measurements at low temperatures, $T < 1$ K, were carried out in LHe dilution refrigerator with low-pass filters, and low-frequency ($f = 19$ Hz) *ac* lock-in techniques with $I = 10$ nA. The tests were performed to ensure the Ohmic regime.

Arrhenius law

In Fig. S1(b-d) we show the effect of the magnetic field on the dependence of R_{sq}/R_N on $1/T$ (on logarithmic scale) for two polycrystalline films, $d = 9.5$ nm (b), and $d = 5.3$ nm (c), and for amorphous film $d = 1.4$ nm (d). The polycrystalline films exhibit clear Arrhenius behavior over the large portions of the data, what indicates flux activation regime. Following the previous analysis of such regime in niobium films by others[3] and us[4], from the slope of linear portions of the Arrhenius plots we may extract the activation energy for the vortex pinning, $\ln(R_{sq}/R_N) = -U(H)/k_BT + K(H)$. Here $U(H)$ is the zero-temperature activation energy and $K(H)$ is the coefficient in the linear T correction. In polycrystalline films the U shows $\ln H$-dependence at high fields ($\mu_0 H \gtrsim 1$ T), and power-law dependence at lower fields; as d decreases and polycrystalline/amorphous boundary is approached the $\ln H$ region expands towards lower fields, down to about 0.1 T. Similar $\ln H$-dependence has been reported previously for thin Nb

films[3,4].

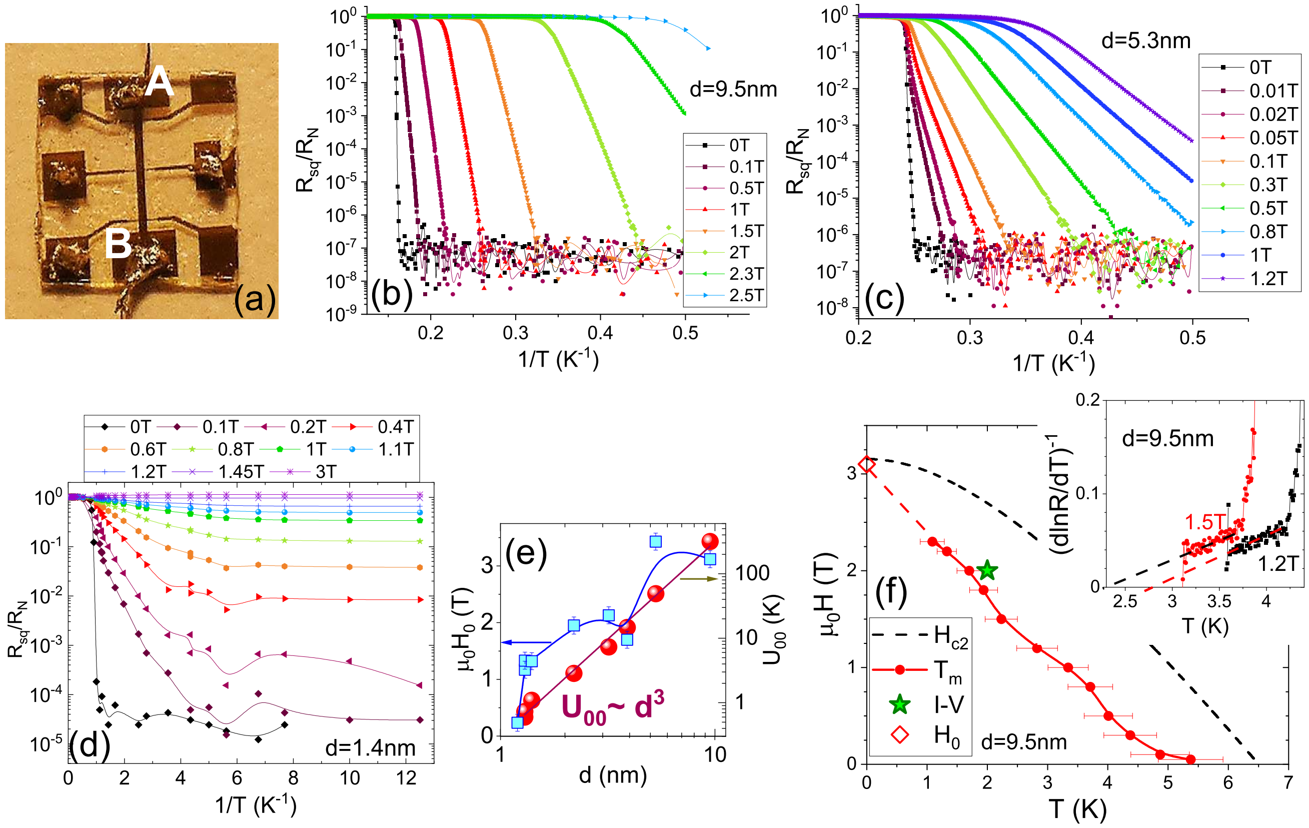

Figure S1. (a) View of Hall-bar structure for the magnetoresistance measurements, with current contacts marked A-B.(b-d) R_{sq}/R_N (on logarithmic scale) versus $1/T$ at labeled magnetic fields for films with (b) $d = 9.5$ nm, (c) $d = 5.3$ nm, and (d) $d = 1.4$ nm. (e) The dependence of the activation energy parameters, H_0 (blue points and line) and U_{00} (red points and line) on the film thickness. (e) The melting line $T_m(H)$ for $d = 9.5$ nm film. The star indicates point determined from I-V measurement. The inset shows the example of the T-dependence of $(d(\ln R)/dT)^{-1}$, used to determine T_m.

In case of amorphous film with $d = 1.4$ nm [Fig. S1(d)] the linear portion of the Arrhenius plots is limited to high temperature range, $T \gtrsim 0.27$ K; at lower T saturation of resistance gradually sets in. The saturation does not shift to higher T as the magnetic field is increased, therefore, we do not believe that magnetic field causes the heating. However, we cannot exclude the possibility that the saturation may be caused by sensitivity of the film to external noise, this issue will be studied in a future investigation. The Arrhenius portions of the plots may be fitted as in the case of polycrystalline films, revealing that the magnetic field dependence of the activation energy in all films is given by $U(H) = U_0 \ln(H_0/H)$, with the parameters H_0 and U_0 shown in Fig.S1(e). The H_0 is the magnetic field at which U extrapolates to zero; it is very close to H_{c2} in polycrystalline films with d=9.5 and 5.3 nm, but drops substantially below H_{c2} on the approach to polycrystalline/amorphous boundary, indicating that the disorder destroys vortex pinning at substantially lower magnetic field. The prefactor U_0 depends on the film thickness as a power law, $U_0 \sim d^3$. Both the logarithmic dependence on the magnetic field, and the dependence of the prefactor on the film thickness are expected in case of collective vortex pinning in thin films[5]. In particular, the prefactor should be proportional to d/Λ_{eff}^2, where Λ_{eff} is the effective penetration depth in thin film,

inversely proportional to d, what results in the observed film thickness dependence. More details on this behavior will be published elsewhere.

In polycrystalline films the flux may remain pinned at the lowest T below melting line, $H_m(T)$, creating zero-resistance vortex glass phase as has been verified in case of thick Nb films[6]. We use standard method to determine this line[7,8], from the fit of linear relation to the dependence of $(d(\ln R)/dT)^{-1}$ versus T, as shown in the inset to Fig.S1(f) for the film with $d = 9.5$ nm. The main figure shows that the melting line extrapolates to the H_0 for $T = 0$. While this method results in considerable error bars, it is clear that the melting line is situated substantially below the $H_{c2}(T)$ line, which is expected in case of ultrathin films. This dependence is further confirmed by the measurement of I-V curves at $T = 2$ K, as indicated by green star in the figure. Similar procedure may be used in all polycrystalline films, and in the thicker amorphous films, down to $d = 2.2$ nm. On the other hand, in case of thinnest amorphous films ($d = 1.3$ or 1.4 nm) we were not able to determine unambiguously such melting line.

Figs.S2(a-b) show the results of I-V measurements for two films, $d = 9.5$ nm measured at $T = 2$ K (a), and $d = 1.3$ nm measured at $T = 50$ mK (b). In (a) we see a behavior typical for superconductor, with zero voltage at low current, and abrupt increase of the voltage once the critical current is reached. In (b), on the other hand, a gradual increase of the voltage starts as soon as the current starts to grow, and this is the case even in the absence of the magnetic field. This suggests that in this film true zero resistive state may be absent. Interestingly, in the vicinity of the critical current a clear evidence of hysteresis is seen, i.e. the abrupt transition to normal state occurs at higher increasing current than the transition to quasi-superconducting state on decreasing current. This hysteretic behavior suggests the possible involvement of the vortex pinning and depinning. Such interpretation is consistent with the fact that on increasing magnetic field hysteresis disappears, since at higher field activation energy decreases, what reduces pinning. We note that we cannot exclude the possibility that hysteresis may arise because of local heating of some areas of the film. However, it may also be related to flux avalanche phenomenon, which prevents vortex pinning on decreasing current at exactly the same current value at which depinning occurs on increasing current. Similar behavior has been observed in thin tantalum films, and interpreted as arising from non-thermal origin[9].

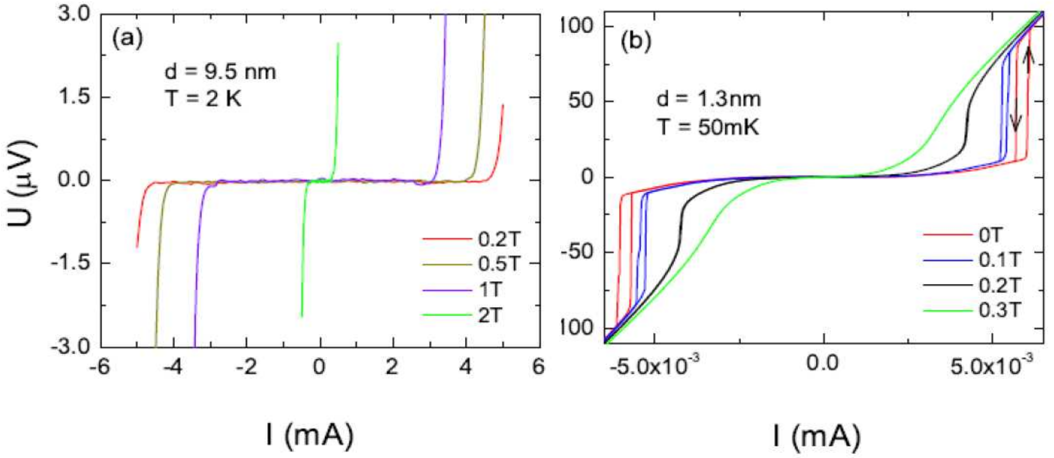

Figure S2. I-V characteristics measured at various magnetic fields for films with $d = 9.5$ nm at $T = 2$ K (a), and $d = 1.3$ nm at $T = 50$ mK (b). In (b) the arrows indicate hysteresis observed for increasing and decreasing current.

Isotherm crossing

Fig. S3 shows the dependence of R_{sq} on B for series of temperatures below 1K for a film with $d = 1.4$ nm. To extract B_c and R_c we determine the crossing points of consecutive isotherms. Starting from high temperatures, when T is reduced from 1 K towards 0.4 K, as shown by grey curved arrow, the crossing points, marked by crosses at the intersections of dashed lines, shift towards lower B. However, when T is reduced below 0.36 K, as shown by black curved arrow, the crossings points (marked by stars at intersections of continuous lines) reach some minimum value of B, and possibly even start to increase slightly (this increase is within experimental error of the measurement).

The inset to Fig. S3 shows the T-dependence of B_c and R_c values determined by this procedure. Within experimental accuracy the B_c and R_c are seen to be constant below the temperature T_0 marked in the figure. The value of B_c, averaged for $T < T_0$, is equal to 1.46 ± 0.01 T.

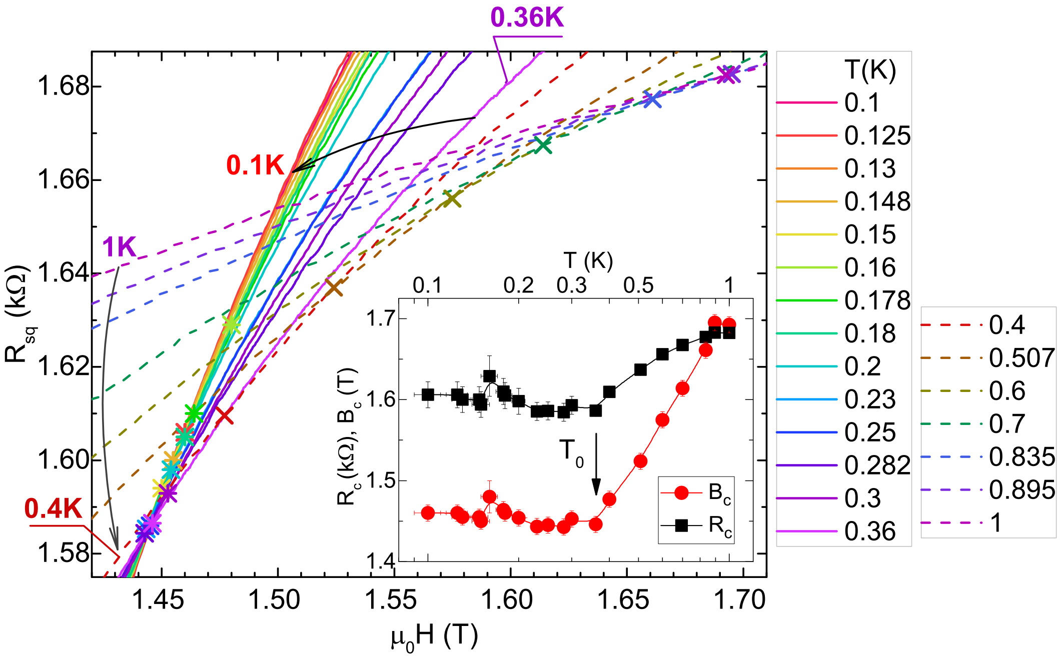

Figure S3. $R_{sq}(B)$ for $d = 1.4$ nm film for series of fixed temperatures, from 1 K to 0.4 K (dashed lines), and from 0.36 K to 0.1 K (continuous lines). The crossings of consecutive isotherms are marked by crosses in high-T range, and by stars in low-T range. The inset shows the T-dependence of B_c (red circles) and R_c (black squares).

Upper critical field

According to WHH theory the upper critical field in the dirty limit can be calculated using the following equation[10]:

$$ln\frac{1}{t} = \left(\frac{1}{2}+\frac{i\lambda_{so}}{4\gamma}\right)\psi\left(\frac{1}{2}+\frac{\bar{h}+\lambda_{so}/2+i\gamma}{2t}\right)+\left(\frac{1}{2}-\frac{i\lambda_{so}}{4\gamma}\right)\psi\left(\frac{1}{2}+\frac{\bar{h}+\lambda_{so}/2-i\gamma}{2t}\right)-\psi\left(\frac{1}{2}\right), \quad \text{(S1)}$$

where $t = T/T_c$, $\gamma \equiv [(\alpha\bar{h})^2 - (\lambda_{so}/2)^2]^{1/2}$ and

$$\frac{\bar{h}}{(-d\bar{h}/dt)_{t=1}} = \frac{\pi^2\bar{h}}{4} = \frac{H_{c2}}{(-dH_{c2}/dt)_{t=1}}. \quad \text{(S2)}$$

When $\alpha = 0$ and $\lambda_{so} = 0$, in the absence of the spin-paramagnetic effect and the weak spin-orbit interaction upper critical field is described by:

$$ln\frac{1}{t} = \psi\left(\frac{1}{2}+\frac{\bar{h}}{2t}\right)-\psi\left(\frac{1}{2}\right), \quad \text{(S3)}$$

than $\mu_0H_{c2}(0) = -A(d\mu_0H_{c2}/dT)_{T_c}T_c$, where the numerical factor A is proportional to $2\Delta/kT_c$.

In BCS weak-coupling superconductors with $2\Delta/kT_c$ = 3.52, A is equal to 0.69. Since the Nb is an intermediate-coupling superconductor, $2\Delta/kT_c$ is larger, so that A is larger as well. It has been shown by Park and Geballe[11] that the value of the $2\Delta/kT_c$ increases with decrease of the film thickness for amorphous Nb-films. Therefore, in our fits we have used the estimates of $2\Delta/kT_c$ reported in Ref.[11]. We have verified that in order to achieve satisfactory fit in the thinnest films, we have to increase the value of $2\Delta/kT_c$ from 3.7 (as in bulk Nb) to 4.5.

In the fitting procedure, we first determine the slope $dH_{c2}/dT|_{T_c}$ from linear fits to $H_{c2}(T)$ line in the vicinity of the T_c. Subsequently, the slope is treated as fixed parameter, while Maki parameters α and λ_{so} are treated as adjustable parameters. The values of $dH_{c2}/dT|_{T_c}$ and $2\Delta/kT_c$ used in the fits, together with Maki parameters estimated from the fits, are shown in Table S1.

d (nm)	$dH_{c2}/dT\|_{T_c}$ (mT/K)	$2\Delta/kT_c$	α	λ_{so}
20	448 (14)	3.9	0	0
9.5	672 (44)	3.9	0.35 (0.1)	0
5.3	1114 (42)	4	1.23 (0.3)	0.5 (0.4)
3.2	2277 (102)	4	2.4 (0.2)	0.7 (0.15)
2.2	2778 (238)	4.1	2.6 (0.3)	0.3 (0.1)
1.4	2648 (124)	4.5	3 (0.1)	0.09 (0.01)
1.3a	2684 (57)	4.5	2.5 (0.1)	0.09 (0.01)

Table S1. The Maki parameters α and λ_{so} estimated from the fit of WHH model to experimental data (the uncertainties are shown in parentheses) for Nb films of thickness d. $2\Delta/kT_c$ are estimates from Ref.[11].

Scaling analysis

Fig. S4(a) shows the plot of $(\partial R_{sq}/\partial B)_{B_c}$ versus $1/T$ for several amorphous films. It is evident that the data do not follow straight lines with the same slope across whole investigated temperature range. Instead, we observe two different T-ranges with distinctly different slopes, marked at the top of the figure by arrows: high-T range ($T \gtrsim 0.5$ K) with larger slope, and low-T range (0.12 K $\lesssim T \lesssim$ 0.35 K) with smaller slope. In addition, at the lowest T ($T \lesssim 0.12$ K) the R_{sq} is saturated (labeled "sat"), so that the slope is zero. When calculating the derivative, we took care to determine it at actual crossings of consecutive isotherms (actual B_c), which shifts with T in high-T range; this shift, however, does not change the slope. Note that the crossover between high-T range and low-T range is not the same for all films, but increases with increasing d; in fact, it occurs approximately at the temperature T_0, below which B_c is constant. The T_0 is marked by small vertical arrows in Fig.S4(a). On the other hand, the B_c remains constant at the crossover between low-T range and saturation region; there is no any apparent signature of this crossover in the B_c value.

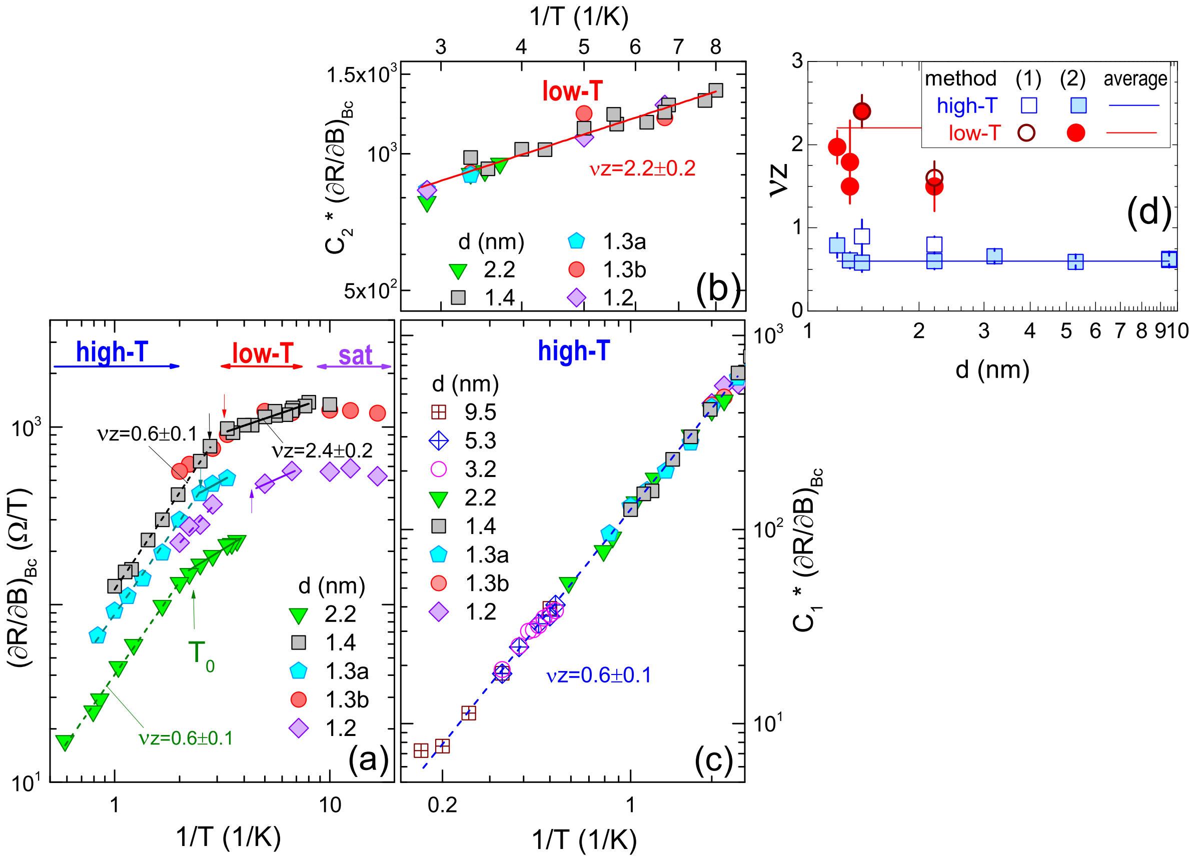

Figure S4. (a) Partial derivative $(\partial R_{sq}/\partial B)_{B_c}$ versus $1/T$ on a log-log scale for several amorphous films with various d. The vertical arrows indicate the temperature T_0, below which B_c is constant. (b-c) Partial derivative data for different films shifted to emphasize the same slope, in low-T (b) and in high-T (c) ranges, respectively. (d) d-dependence of νz and "apparent νz" for high-T (blue) and low-T (red) ranges, determined by method (1) (open points) and method (2) (full points), and averaged for all films (lines).

Interestingly, in both high-T, and low-T ranges the slopes for many different films are similar. To emphasize this point, we plot in Figs.S4(b) and S4(c) the data for low-T and high-T ranges, respectively,

after shifting the data for different films vertically, so all of them overlap the data for the film with $d = 1.4$ nm (this amounts to multiplying data for each film by a different constant, C_1 or C_2 for high-T or low-T range, respectively). In the high-T range we include, in addition, the data for polycrystalline films - it is seen that they show exactly the same slope as the data for amorphous films. From the slope of straight line, fitted to all data for different amorphous films in low-T range, average critical exponent is determined: $\nu z = 2.2 \pm 0.2$. Similar procedure applied to high-T range gives the average inverse of the slope equal to 0.6 ± 0.1. While in polycrystalline films this quantity may be identified as critical exponent, in case of amorphous films this is less likely, because the B_c and the R_c are not constant across the high-T range, so that one parameter scaling may be invalid - therefore, we call it "apparent critical exponent".

We summarize the scaling analysis results in Fig.S4(d), where points show the d-dependence of νz and "apparent νz", extracted separately for each film using method (1) and (2) (as defined in the main paper), while lines show the average values as fitted to data in Figs.S4(b) and S4(c). The high-T values are all similar, independent of the sample or the method used. On the other hand, low-T values of νz extracted separately are considerably scattered, between 1.5 and 2.4, possibly due to small T-range of the mK measurements in case of some films. Nevertheless there is no doubt that the low-T exponent in the amorphous films exceeds 1.

Superconducting fluctuations

We first compare our experimental data to formulas provided by Galitski and Larkin[12], and we conclude that reasonably good fit may be achieved. It is restricted, however, to low-T, and to the vicinity of H_{c2}, i.e. $h \lesssim 2.5$. The data are fitted by the following equations:

$$\Delta G = G - G_n = \frac{e^2}{\pi^2 \hbar}[\alpha(t) I_\alpha(t,h) + \beta(t) I_\beta(t,h)], \tag{S4}$$

$$I_\alpha(t,h) = \ln\frac{r}{h} - \frac{1}{2r} - \psi(r), \tag{S5}$$

$$I_\beta(t,h) = r\psi\prime(r) - \frac{1}{2r} - 1, \tag{S6}$$

where $r = h/3.562t$, ψ is digamma function, and G_n is the normal state resistance. In these equations ΔG is in units of $G_0 = e^2/2\pi^2\hbar$, $t = T/T_{c0}$, and $h = H/H_{c2}(0) - 1$. We use $H_{c2}(0)$ determined from the WHH expressions. Parameters for the fit are α, β and G_n (G_n is close to G value at maximum h). In addition, in order to fit all the data with minimal variations on the other parameters it has been necessary to allow T to vary slightly, by about ± 0.015 K. The fitting process goes as follows. First, a set of acceptable parameters is obtained for a single $\Delta G(t,h)$ versus h curve. Using these parameters as a starting point, individual fits for $\Delta G(t,h)$ versus h curves were done at different temperatures, yielding α, β and G_n for each value of t. After fitting all the curves, $\alpha(t)$, $\beta(t)$ and $G_n(t)$ were kept constant and T_{c0} was varied in order to improve the fit of all $\Delta G(t,h)$ versus h curves simultaneously. Deviations were then addressed by adjusting each curve's T.

The results of the fits, together with fitting parameters, are displayed in Fig. S5. The fitting becomes worse as t is increased, and diverges from the data at $h > 2.5$. We note that, while these formula do not provide the ideal fits, the essential result of negative ΔG is well reproduced.

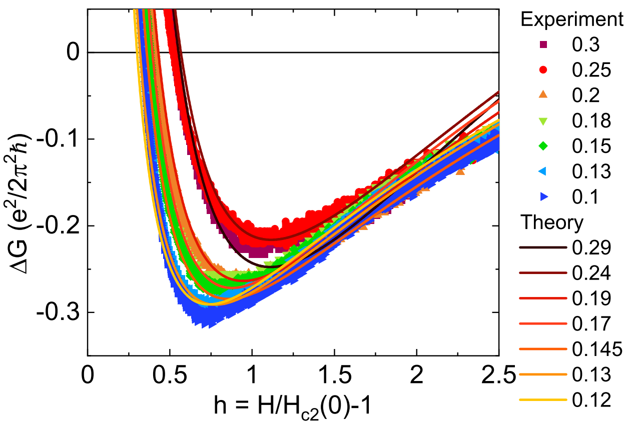

T(K)	Fitted T(K)	α	β	G_n (in units of G_0)
0.1	0.12	-0.3	1.233	43.63
0.13	0.13	-0.313	1.233	43.66
0.15	0.145	-0.326	1.267	43.74
0.18	0.17	-0.393	1.333	43.76
0.2	0.19	-0.4	1.333	43.84
0.25	0.24	-0.487	1.433	44
0.3	0.29	-0.567	1.367	44.12

Figure S5. Comparison of experimental data (points) and theory of Ref.[12](lines); parameters of the fit are listed in the table.

Next, we compare experimental data to theory of Glatz et al.[13]. In order to avoid tedious calculations, we use simplified approach, fitting to the data asymptotic functions provided by theory for several distinct domains in the phase diagram, as defined by Glatz et al.[13]. These are the following domains: domain IV (region of quantum fluctuations), domain V (quantum to classical region), domain VI-VII (classical, strong fields), and domain IX (high magnetic fields). We disregard as insignificant all residual parts of the order of $(t/h)^2$. Since in our experiment the temperature is not zero, but finite, even at the lowest T the increase of the field drives the system first through domain V, and subsequently, at higher field, through domain IV. Accordingly, we break the range of the data for the fittings into three different regions: (1) low-T, low-h (2) low-T, high h (3) high-T. The first two regions cover the same T-range ($T \leq T_0$), but are distinguished by the h value. In region (1) we use a combination of asymptotic expressions from domains V and IV to fit the data (the first one gives positive, and the second negative contribution), while in region (2) it is enough to use expressions from domain IV. Finally, the data in the region (3) are well fitted by combination of expressions from domains VI-VII and IX. Note that when expressions from two domains are included, it is necessary to include additional, field-independent parameter, in order to offset the excess asymptotic value of ΔG. Accordingly, we use the following functions to fit the data:

$$\Delta G = A\frac{4}{3}\gamma_E\frac{t}{h} - B\frac{4}{3}\ln\left(\frac{1}{h}\right) + C, \qquad 0.1 \geq t \geq 0.4 \tag{S7}$$

$$\Delta G = A\frac{\pi^2}{3}\frac{0.88H_R t}{h+1-H_R} + B\frac{4}{3}\ln\left(\ln\left(0.88(h+1)\right)\right) + C, \qquad 0.4 \geq t \geq 1 \tag{S8}$$

Here $\gamma_E = 1.78107$ is Euler's constant, ΔG is in units of $G_0 = e^2/2\pi^2\hbar$, $t = T/T_{c0}$, and $h = H/H_{c2}(0) - 1$. The factor 0.88 comes from the definition of dimensionless magnetic field ($0.88H/H_{c2}(0)$), and in case of thin Nb films replaces the usual factor of 0.69 (valid for weak-coupling superconductor), as discussed in case of WHH equations. Finally, the constant H_R is given by $H_R = H_{c2}(T)/H_{c2}(0)$; it deviates slightly from 1 for temperatures close to T_{c0}, but it approaches 1 on cooling down to $t = 0.5$. We use $T_{c0} = 1$ K, and $H_{c2}(0)$ determined from the WHH expressions. A, B and C are fitting parameters. Parameter C is a h-independent constant, added in order to offset the excessive background resulting from the inclusion of asymptotic expressions from two domains, or/and in order to account for any deviation of the $G_{8.5}$ from the real normal-state conductivity.

Region(1) low t, low h				Region(2) low t, high h				Region(3) high t				
t	A	B	C	t	A	B	C	t	A	B	C	H_R
0.4	1.00395	0.42987	-0.98666	0.4	0.2051	0.15235	-0.35313	1.0	0.93107	0.6723	-0.92699	0.86285
0.36	0.98062	0.42572	-0.93077	0.36	0.06702	0.11323	-0.24534	0.9	0.98602	0.74852	-0.95191	0.89817
0.3	0.99462	0.48689	-0.93406	0.3	-0.35465	0.06294	-0.07782	0.84	0.91622	0.68567	-0.87159	0.92751
0.25	1.07951	0.43805	-0.85121	0.25	-0.15384	0.09045	-0.18177	0.7	0.90785	0.7013	-0.81443	0.96617
0.2	1.18139	0.45643	-0.82532	0.2	-0.43257	0.08037	-0.11787	0.6	0.92151	0.70602	-0.77439	0.98808
0.18	1.22542	0.47135	-0.78517	0.18	-0.57565	0.06247	-0.06823	0.51	0.93548	0.67683	-0.71094	1.0
0.15	1.50561	0.48047	-0.80343	0.15	-0.51689	0.08416	-0.12723	0.4	1.03512	0.70744	-0.68762	1.0
0.13	1.61026	0.4797	-0.77544	0.13	-0.81738	0.06542	-0.07434					
0.1	2.09809	0.48882	-0.78445	0.1	-0.96136	0.073	-0.10147					

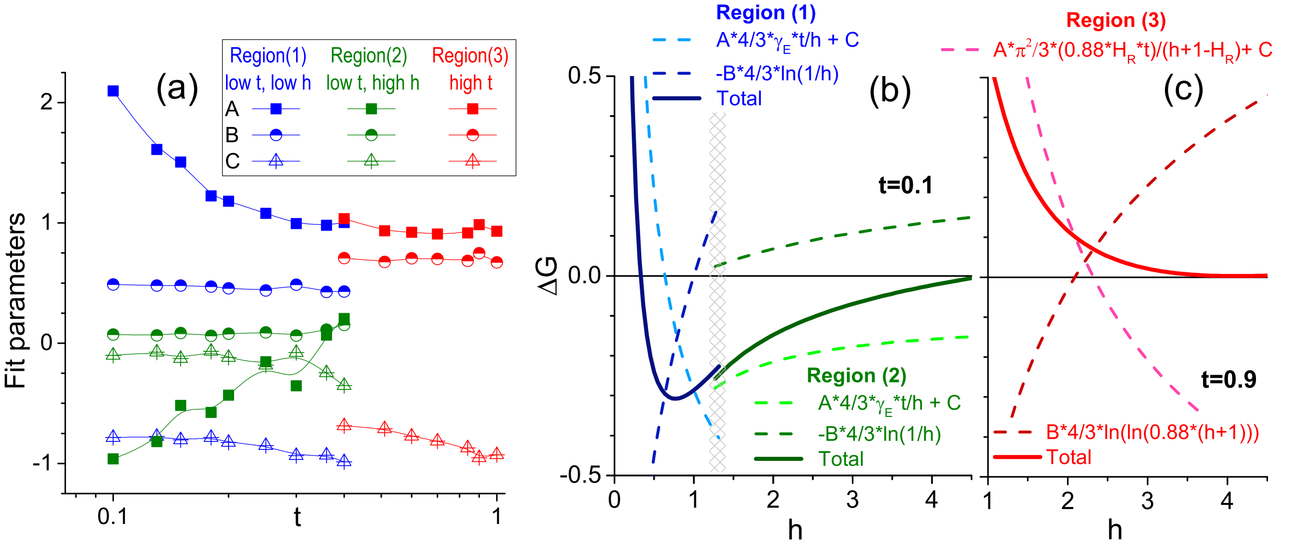

Figure S6. Table: parameters of the fit of asymptotical expressions from Glatz et al.[13,14] to experimental data. (a) Fitting parameters versus t for different fit regions. (b)-(c) The h-dependence of different parts (dashed lines) and of total ΔG curve (continuous line) of Eq.S7 (b), and of Eq.S8 (c).

The Eq.S7 describes low-t data with different set of parameters for low-h (region (1)) and high-h (region (2)), while Eq.S8 is used to fit high-t data (region (3)). At the top of Fig.S6 we show the table with all sets of parameters for different regions; the T-dependence of these parameters is plotted in Fig.S6(a). In parts (b) and (c) of this figure we show the examples of h-dependencies of different terms of both equations, and the total curves $\Delta G(h)$, for low-t regions (1) and (2), and for high-t region (3), respectively.

In the region (2) (low-t, high-h) the system remains exclusively in the domain IV. At the lowest T the A parameter is negative, so that first part of Eq.S7 gives negative contribution to fluctuation conductivity; the contribution of the second part is positive, while the constant C is small and negative. This results in the total negative contribution, which approaches zero at large h. On the other hand, in the region (1) (low-t, low-h), around the minimum on the $\Delta G(h)$ curve) the two terms in Eq.S4 result from two domains: domain V (quantum to classical region, it gives positive contribution to first term) and domain IV (quantum region, it gives negative contributions to both terms) - this is because in our experiment we keep t constant, and increase h, therefore, the system is driven first through domain V, and subsequently through domain IV. Because of contributions from two domains the offset C is sizeable. The resulting total $\Delta G(h)$ curve describes very well the minimum, which appears in region (1). Note that the boundary between regions (1) and (2) is t-dependent, and shifts from h=1.24 up to h=2.2 when t is increased from

0.1 up to 0.4.

In Eq.S8 the first term (t- and h-dependent) comes from formula for domain VI-VII. It is positive for small h, describing well small-h behavior of ΔG. However, to obtain the correct dependence for large h we have to include, in addition, second term, which is h-dependent part of the formula from domain IX (t- and h-independent terms from domain IX are included in the constant C). Note that parameter A extrapolates smoothly from region (3) into region (1) at $t = 0.4$. In fact, the experimental data for $T = 0.4$ K are reasonably well fitted both by Eq.S7 and by Eq.S8, confirming the fact at that this temperature a crossover occurs from quantum to classical fluctuations.

The final curves for ΔG for the three different regions of the fits are compared with experimental data for $d = 1.4$ film in Fig. 3(b) in the main paper. Interestingly, the negative contribution exists also in case of polycrystalline film with $d = 9.5$ nm, although it is far less spectacular. Fig.3(c) (main paper) shows the $\Delta G = G - G_9$ (G_9 is the conductivity at 9 tesla) for this film. Since the H_{c2} is larger in this film, the data are limited to smaller h-range, so we can test the behavior at two lowest measured temperatures only (2 and 3 K); in addition, the data are more noisy. Nevertheless, it is clear that the minimum appears; moreover, the data may be described by the expressions from region (1) (fits of expressions from region (3) are unsuccessful).

References

1. Zaytseva, I. *et al*. Negative Hall coefficient of ultrathin niobium in Si/Nb/Si trilayers. *Phys. Rev. B* **90,** 060505 (2014).
2. Demchenko, I. N. *et al*. Use of XPS to clarify the Hall coefficient sign variation in thin niobium layers buried in silicon. *Appl. Surf. Science* **399,** 32 (2017).
3. Hsu, J. W. P. and Kapitulnik, A. Superconducting transition, fluctuation, and vortex motion in a two-dimensional single-crystal Nb film. *Phys. Rev. B* **45,** 4819 (1992).
4. Zhu, Leyi Y., Cieplak, M. Z., and Chien C. L. Tunable phase diagram and vortex pinning in a superconductor-ferromagnet bilayer. *Phys. Rev. B* **82**, 060503(R) (2010).
5. Jensen, H. J. *et al*. Vortex Fluctuations, Negative Hall Effect, and Thermally Activated Resistivity in Layered and Thin-Film Superconductors in an External Magnetic Field. *Europhys. Lett.* **20**, 463 (1992).
6. Villegas, J. E. and Vicent, J. L. Vortex-glass transitions in low-Tc superconducting Nb thin films and Nb/Cu superlattices. *Phys. Rev. B*. **71**, 144522 (2005).
7. Safar, H. *et al*., SQUID Picovoltometry of Single Crystal $Bi_2Sr_2CaCu_2O_{8+\delta}$: Observation of the Crossover from High-Temperature Arrhenius to Low-Temperature Vortex-Glass Behavior. *Phys. Rev. B*. **68**, 2672 (1992).
8. Lee H.-S. *et al*., Magnetic-field-induced crossover of vortex-line coupling in SmFeAsO0.85 single crystal. *Phys. Rev. B*., **82**, 104523 (2010).
9. Y. Qin, C. L. Vicente, and J. Yoon, Magnetically induced metallic phase in superconducting tantalum films. *Phys. Rev. B*., **73**, 100505 (2006); Y. Seo, *et al*., Origin of nonlinear transport across the magnetically induced superconductor-metal-insulator transition in two dimensions, *Phys. Rev. Lett.* **97**, 057005 (2006).
10. Werthamer, N. R., Helfand, E. and Hohenberg, P. C. Temperature and purity dependence of the superconducting critical field, H_{c2}. III. Electron spin and spin-orbit effects. *Phys. Rev.* **147,** 295 (1966).

11. Park, S. I. and Geballe, T. H. Superconducting Tunneling in Ultrathin Nb Films. *Phys. Rev. Lett*. **57,** 901 (1986).

12. Galitski, V. M., and Larkin, A.I. Superconducting fluctuations at low temperature. *Phys. Rev. B* **63**, 174506 (2001).

13. Glatz, A., Varlamov, A. A., and Vinokur, V. M. Fluctuation spectroscopy of disordered two-dimensional superconductors. *Phys. Rev. B* **84**, 104510 (2011).

14. Varlamov A.A., Galda A., and Glatz A. Fluctuation spectroscopy: From Rayleigh-Jeans waves to Abrikosov vortex clusters. *Rev. Mod. Phys.* **90**, 015009 (2018).
